# BMP-7 ameliorates partial epithelial-mesenchymal transition by restoring SnoN protein level via Smad1/5 pathway in diabetic kidney disease

**DOI:** 10.1038/s41419-022-04529-x

**Published:** 2022-03-21

**Authors:** Wei Peng, Xingcheng Zhou, Tingting Xu, Yanwen Mao, Xiaohuan Zhang, Huiming Liu, Luqun Liang, Lingling Liu, Lirong Liu, Ying Xiao, Fan Zhang, Shuang Li, Mingjun Shi, Yuxia Zhou, Lei Tang, Yuanyuan Wang, Bing Guo

**Affiliations:** 1 Guizhou Provincial Key Laboratory of Pathogenesis and Drug Research on Common Chronic Diseases, Guizhou Medical University, Guiyang, China; 2 Department of Pathophysiology, Guizhou Medical University, Guiyang Guizhou, 550025 China; 3 Clinical Laboratory Center, The First People Hospital of Yueyang, Yueyang Hunan, 414000 China; 4 School of Medicine and Holistic Integrative Medicine, Nanjing University of Chinese Medicine, Nanjing 210046 Jiangsu, China; 5 Department of Pathophysiology, Guizhou Medical Hospital, Guiyang Guizhou, 550002 China; 6 Guizhou Provincial Engineering Technology Research Center for Chemical Drug R&D, Guizhou Medical University, Guiyang Guizhou, 550025 China; 7 State Key Laboratory of Functions and Applications of Medicinal Plants, Guizhou Medical University, Guiyang Guizhou, 550025 China

**Keywords:** Molecular biology, Post-translational modifications

## Abstract

Tubulointerstitial fibrosis (TIF) is involved in the development of diabetic kidney disease (DKD). Transforming growth factor β1 (TGF-β1) is involved in the extensive fibrosis of renal tissue by facilitating the partial epithelial-mesenchymal transition (EMT), increasing the synthesis of extracellular matrix (ECM), inhibiting degradation, inducing apoptosis of renal parenchyma cells, and activating renal interstitial fibroblasts and inflammatory cells. Recent studies indicated that bone morphogenetic protein-7 (BMP-7) upregulated the expression of endogenous SnoN against renal TIF induced by TGF-β1 or hyperglycemia. Nevertheless, the mechanisms underlying the BMP-7-mediated restoration of SnoN protein level remains elusive. The present study demonstrated the increased expression of BMP-7 in diabetic mellitus (DM) mice by hydrodynamic tail vein injection of overexpressed BMP-7 plasmid, which attenuated the effects of DM on kidney in mice. Partial tubular EMT and the accumulation of Collagen-III were resisted in DM mice that received overexpressed BMP-7 plasmid. Similar in vivo results showed that BMP-7 was competent to alleviate NRK-52E cells undergoing partial EMT in a high-glucose milieu. Furthermore, exogenous BMP-7 activated the Smad1/5 pathway to promote gene transcription of *SnoN* and intervened ubiquitination of SnoN; both effects repaired the SnoN protein level in renal tubular cells and kidney tissues of DM mice. Therefore, these findings suggested that BMP-7 could upregulate *SnoN* mRNA and protein levels by activating the classical Smad1/5 pathway to refrain from the partial EMT of renal tubular epithelial cells and the deposition of ECM in DKD-induced renal fibrosis.

## Introduction

Diabetic kidney disease (DKD) involves glomeruli and tubules, and the common pathological changes include glomerulosclerosis and tubulointerstitial fibrosis (TIF), which eventually develop into renal sclerosis [1, 2]. TIF features interstitial matrix deposition, inflammation, fibroblast activation, microvascular rarefaction, and tubular cell loss. Renal tubular epithelial cells (RTECs), as the main renal parenchyma cells, activate several profibrotic signaling pathways after sustained-stimulation by hyperglycemia, resulting in TIF-associated functional disorders [3]. Hitherto, transforming growth factor β1 (TGF-β1) is considered as the key cytokine to promote renal TIF in DKD [4, 5]. It also partakes in fibrosis of renal tissue by facilitating the partial epithelial-mesenchymal transition (EMT), increasing the synthesis of extracellular matrix (ECM), inhibiting degradation, inducing apoptosis of renal parenchyma cells, as well as activating renal interstitial fibroblasts and inflammatory cells.

Bone morphogenetic protein-7 (BMP-7) is a member of the TGF-β superfamily, which is mainly expressed in podocytes and tubular epithelial cells in the kidney [6, 7]. Intracellular signal transduction of BMP-7 includes a classical pathway mediated by Smad proteins and a nonclassical pathway being independent of Smad proteins. Briefly, BMP-7 rapidly activates Smad1/5/8, which then binds to Smad4 in the cytoplasm to form a transcriptional complex. This complex is a structural requisite for nuclear translocation and mediating transcriptional activation of target genes [6, 8]. BMP-7 also activates a number of nonclassical pathways, such as extracellular signal-regulated kinase (ERK), C-Jun amino-terminal kinase (JNK), and p38 mitogen protein kinase (p38MAPK) [8–10]. Our previous results and other studies have confirmed that hyperglycemia decreased the mRNA and protein levels of BMP-7 in RTECs, and it was negatively correlated with renal fibrosis [10–12]. The effects of BMP-7 on mesenchymal-epithelial transition (MET) during renal development and the inhibitory functions on TGF-β1-mediated fibrosis in renal diseases have markedly attracted researchers’ attention [8, 12, 13]. Nonetheless, how BMP-7 antagonizes the fibrogenic effects of the TGF-β1 signaling pathway needs to be elucidated. Previous studies demonstrated that rhBMP-7 upregulated the expression of endogenous SnoN against renal TIF induced by TGF-β1 or hyperglycemia [12, 14].

Transcriptional co-inhibitor Ski-related novel protein N (SnoN), a major negative regulator of TGF-β1/Smad signaling pathway, interacts with Smad2/3/4 complex in the cytoplasm to inhibit its nuclear translocation. Moreover, SnoN interferes with the interaction between activated Smad complex and DNA, blocks the interaction of Smad2/3 with transcriptional co-activating factor, and recruits a transcriptional co-inhibitor, thereby regulating the biological effects of TGF-β1 by suppressing the transcriptional activation of the target gene [15, 16]. A number of studies also showed that in renal fibrosis caused by a unilateral ureteral obstruction (UUO) or DKD, the level of SnoN protein decreased gradually, while the hyperactivity of TGF-β1/Smad signaling pathway was persistent, accompanied by the phenotypic changes of RETCs and the deposition of ECM in the interstitium [17–19]. Further studies indicated that the expression level of SnoN was tightly regulated with respect to transcriptional activation and protein stability [19]. In addition, TGF-β1 strongly upregulates the expression of *SnoN* mRNA. On the other hand, the high level of TGF-β1 protein induces rapid and significant degradation of SnoN protein [17, 19]. Nevertheless, the mechanism involved in the role of BMP-7 in restoring SnoN protein level, whether BMP-7 merely promotes the transcription of *SnoN* gene or it is accompanied by preventing the degradation of *SnoN* protein, and whether this effect is proceeded by activating intracellular Smadl/5 pathway remains to be explored. Herein, we asserted that BMP-7 upregulates *SnoN* mRNA and protein levels by activating the classical Smad1/5 pathway to refrain from the partial EMT of RTECs and the deposition of ECM in diabetes-induced renal fibrosis.

## Results

### Exogenous BMP-7 reduces the effect of diabetes on kidney in mice

**Fig. 1:**
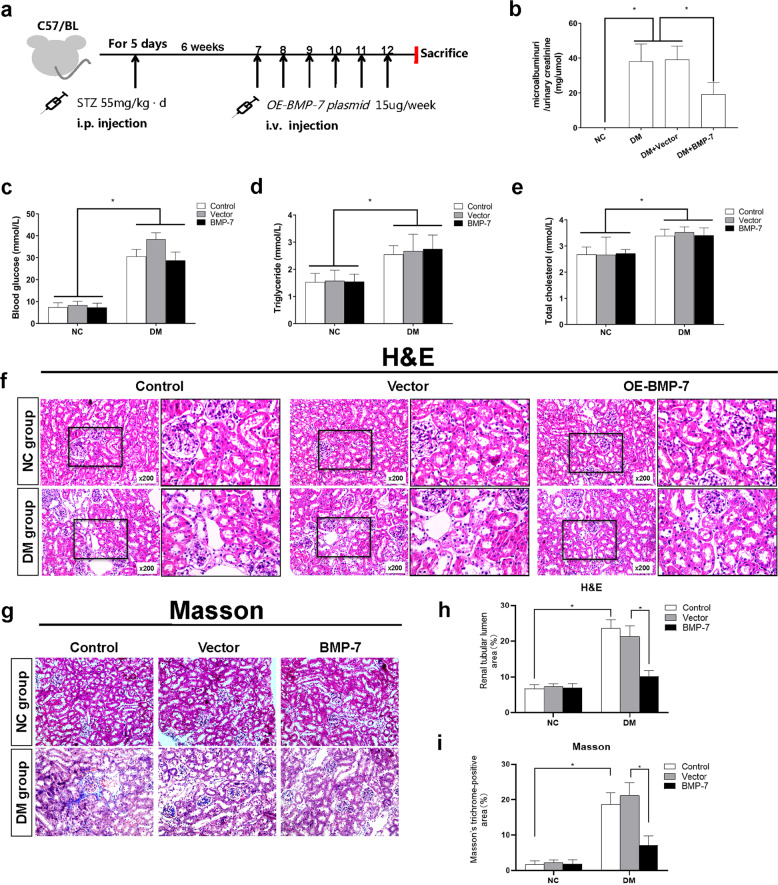
BMP-7 ameliorated renal fibrosis in diabetic mice. **a** Rescue experiment design. At 6 weeks after the diabetic mice model had been successfully established, *OE-BMP-7* plasmid was injected i.v. every week for 6 consecutive weeks, and mice were killed at the end of 12 weeks.**b** Urinary microalbumin/urinary creatinine level in NC + Control group and the DM group (DM + Control, DM + Vector, DM + BMP-7) mice,*P < 0.05 (*n* = 6). **c** Blood glucose, **d** Triglyceride, **e** Total cholesterol levels in the NC group (NC + Control, NC + Vector, NC + BMP-7) and DM group (DM + Control, DM + Vector, DM + BMP-7) mice,*P < 0.05 (*n* = 6). Histological changes of kidneys in the NC group and DM group **f** (hematoxylin-eosin staining), **g** (Masson’s trichrome staining)black box indicates the enlarged area of details for fibrosis(magnification ×400). **h** Renal tubular lumen area was analyzed in each group from six random fields (200×), *P < 0.05 (*n* = 6). **i** Positive staining density of Masson’s trichrome staining was analyzed in each group from six random fields (200×), *P < 0.05 (*n* = 6).

To confirm the antifibrotic mechanisms of BMP-7, we injected overexpressing-hBMP-7 (*OE-BMP*-*7*) plasmid (Supplementary Fig. 1a) through the hydrodynamic tail vein injection to elevate BMP-7 expression in the kidney. Firstly, as illustrated in Fig. 1a, mice were injected with STZ for 5 days to establish the diabetic model. After the diabetic mice model had been successfully established for 6 weeks, the animals were injected with vehicle, vector, or *OE-BMP-7* plasmid, respectively, by i.v. every week, for 6 weeks, and the animals were sacrificed at the end of 12 weeks. Secondly, we determined the expression of BMP-7 by Western blotting and IF staining. The data indicated that BMP-7 protein expression was downregulated in diabetic mice, while it was upregulated by injection of the *OE-BMP-7* plasmid (Supplementary Fig. 1b–d).

Urinary microalbumin/urinary creatinine, blood glucose, TGs, and TC were significantly induced in diabetic mice (Fig. 1b–e). Besides, no significant difference was detected in these indexes among diabetic mice by injection of vector plasmid. However, after injection of the *OE-BMP-7* plasmid, the level of urinary microalbumin/creatinine decreased significantly, suggesting that BMP-7 has a protective effect on diabetic renal injury. This result was supported by the histopathological changes. HE and Masson’s trichrome staining methods showed an increase in the mesangial matrix, the thickness of the glomerular basement membrane, and hypertrophy of glomeruli in 12-week-old diabetic mice. Moreover, a subset of tubules was atrophied or lost, and the inflammatory cells increased and infiltrated into widened tubulointerstitium. The pathological changes were alleviated after injection of the OE-BMP-7 plasmid (Fig. 1f, i).

### Exogenous BMP-7 restrains DKD progression by resisting partial tubular EMT and the accumulation of Collagen-III in diabetic mice and renal epithelia

**Fig. 2:**
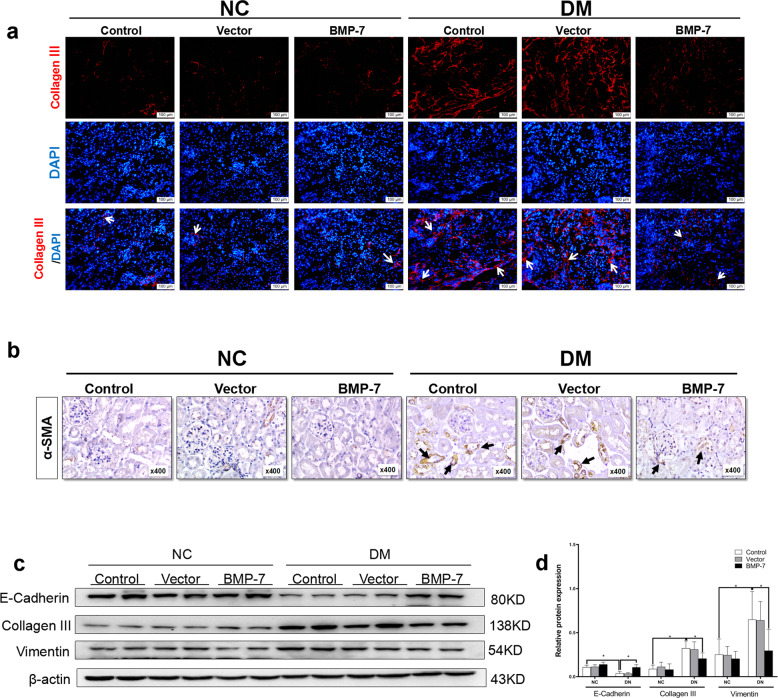
BMP-7 inhibited the expression of Collagen-III and tubular epithelial to mesenchymal transition in kidneys of diabetic mice. **a** Representative micrographs show immunofluorescence staining of Collagen-III in different groups. DAPI staining(blue) represents cell nucleus, Bar = 100 um. Arrows (→) indicate positive expression. **b** Representative micrographs show immunohistochemical staining of α-SMA in different groups (magnification ×400). Arrows (→) indicate positive expression. Representative **c** Western blotting and **d** quantitative data were revealed the expression of E-cadherin, Collagen- III, Vimentin, in the kidney tissues in different groups, *P < 0.05 (*n* = 6).

Next, we examined the effects of exogenous BMP-7 on diabetic mice. As depicted in Fig. 2a, Collagen-III deposition was largely confined to the tubulointerstitium in the kidneys of diabetic mice and reduced in the kidneys of diabetic mice injected with *OE-BMP-7* plasmid (Fig. 2a and Supplementary Fig. 1e). Moreover, positive α-SMA expression was detected in the renal tubule and around the blood vessels in the tubulointerstitium of diabetic mice (Fig. 2b and Supplementary Fig. 1f). Consistent with the immunohistochemical analysis, the results of Western blotting revealed that the expressions of Vimentin and Collagen-III increased, while the expression of E-cadherin decreased in the kidneys of diabetic mice, whether in the control group or in the vector group (Fig. 2c, d). Conversely, BMP-7 suppressed diabetes-induced α-SMA and Collagen-III protein expression and restored E-cadherin expression in vivo.

**Fig. 3:**
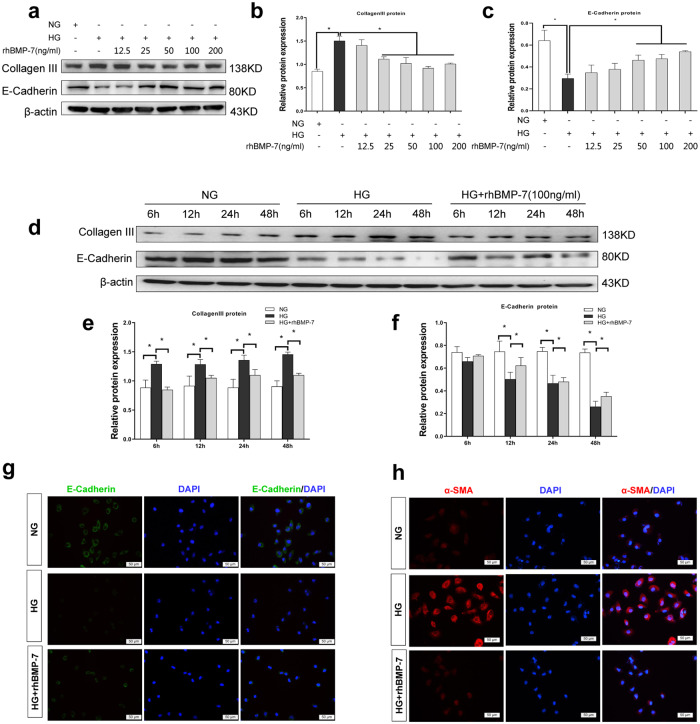
BMP-7 inhibited high glucose-mediated Collagen-III expression and preserved cell phenotype of NRK-52E cell. Representative **a** Western blot and quantitative data were revealed the expression of **b** Collagen-III, **c** E-cadherin, in NRK-52E cells under normal-glucose condition, high glucose condition with either vehicle or recombinant human BMP-7(rhBMP-7) at different dosages,*P < 0.05 (*n* = 3). Representative **d** Western blot and quantitative data were revealed the expression of **e** Collagen-III, **f** E-cadherin, in NRK-52E cells under normal-glucose condition, high glucose condition with rhBMP-7(100 ng/ml) at different time, *P < 0.05 (*n* = 3). **g** Representative micrographs show immunofluorescence staining of E-cadherin in different groups. DAPI staining(blue) represents cell nucleus, Bar = 50 um. **h** Representative micrographs show immunofluorescence staining of α-SMA in different groups. DAPI staining(blue) represents cell nucleus, Bar = 50 um.

To investigate the influences of BMP-7 on proximal tubular epithelial cells during the development of DKD, NRK-52E cells were cultured with a high glucose medium and co-treated with different concentrations of recombinant human BMP-7 (rhBMP-7)(12.5, 25, 50, 100, and 200 ng/mL) for 48 h, or co-cultured with 100 ng/mL rhBMP-7 at different time points. Consistently, the significant inhibition of Collagen-III and recovery of E-cadherin were detected in NRK-52E cells co-treated with rhBMP-7 in a dose- and time-dependent manner (Fig. 3a–f). Furthermore, the results of Western blotting and IF staining revealed that treating NRK-52E cells with rhBMP-7 suppressed high glucose-induced α-SMA protein expression and restored E-cadherin expression (Fig. 3g, h and Supplementary Fig. 1g, h). These findings indicated that BMP-7 was competent to alleviate partial EMT during DKD.

### BMP-7 inhibits partial EMT via upregulation of SnoN

**Fig. 4:**
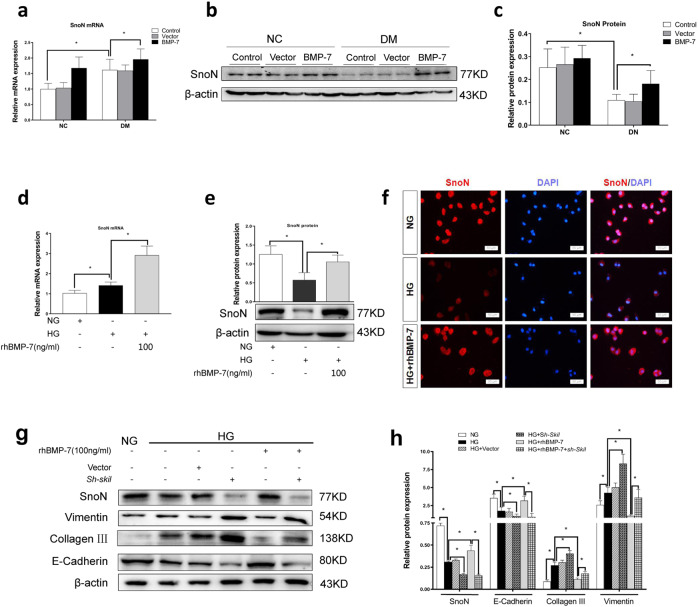
BMP-7-restored SnoN protein countered renal fibrosis after diabetes. **a** Graphical presentations show the relative abundance of *SnoN* mRNA after normalization with *β-actin* mRNA analyzed by RT-qPCR in the kidney tissues in different groups, *P < 0.05 (*n* = 6). **b** Western blotting and **c** quantitative data represent the expression of SnoN protein in the kidney tissues in different groups, *P < 0.05 (*n* = 6). **d** Graphical presentations show the relative abundance of *SnoN* mRNA after normalization with *β-actin* mRNA analyzed by RT-qPCR in NRK-52E cells under different conditions, *P < 0.05 (*n* = 3). **e** Western blotting and quantitative data represent the expression of SnoN protein in NRK-52E cells under different conditions, *P < 0.05 (*n* = 3). **f** Representative micrographs show immunofluorescence staining of SnoN in NRK-52E cells after incubation in different conditions. DAPI staining (blue) represents cell nucleus, Bar = 50 um. Knockdown of SnoN impaired the renoprotection of BMP-7 in NRK-52E cells under high-glucose conditions. **g** Western blotting and **h** quantitative data represent the expressions of E-cadherin, Collagen-III, and Vimentin in NRK-52E cells under different conditions, *P < 0.05 (*n* = 3).

SnoN is a negative regulator of the TGF-β1/Smad signaling pathway. In the kidney, inconsistent with the downregulation of SnoN protein, *SnoN* mRNA level significantly increased in response to diabetes in vehicle- and vector-treated mice. Additionally, the synergistic effects of BMP-7 and high glucose were observed in kidneys of *OE-BMP-7* plasmid-treated mice, wherein *SnoN* mRNA expression was further upregulated and the protein expression was repaired compared to the Vector group in diabetic mice (Fig. 4a–c).

The results of RT-qPCR indicated a higher level of *SnoN* mRNA, and the results of Western blotting demonstrated that BMP-7 induced SnoN protein expression in NRK-52E cells exposed to high glucose medium co-treated with exogenous rhBMP-7 (Fig. 4d, e). As shown in Fig. 4f, IF staining indicated the reduced SnoN protein level in NRK-52E cells under high-glucose conditions and augmented the expression of SnoN protein in NRK-52E cells under high-glucose co-treatment with rhBMP-7 (The statistical results are shown in Supplementary Fig. 2a). Moreover, IF staining verified that SnoN was localized in either cytoplasm or nucleus in renal epithelia, demonstrating that SnoN protein comes into effect in the cytoplasm and nucleus.

Then, we knocked down SnoN expression by transfecting *sh-Skil* plasmid (Supplementary Fig. 2b, c), followed by treatment with high-glucose and rhBMP-7 (100 ng/mL). As displayed in Fig. 4g, h, co-treatment with BMP-7 inhibited NRK-52E cells from a high glucose-induced increase in Collagen-III and Vimentin and promoted E-cadherin reexpression. However, the protective effect of BMP-7 on renal tubules was impaired almost after a decrease in SnoN protein by transfecting the *sh-Skil* plasmid. The results indicated that BMP-7 could inhibit partial EMT mainly via SnoN.

### BMP-7 preserves SnoN protein level via activation of Smad1/5 pathway based on gene transcription and protein stability

**Fig. 5:**
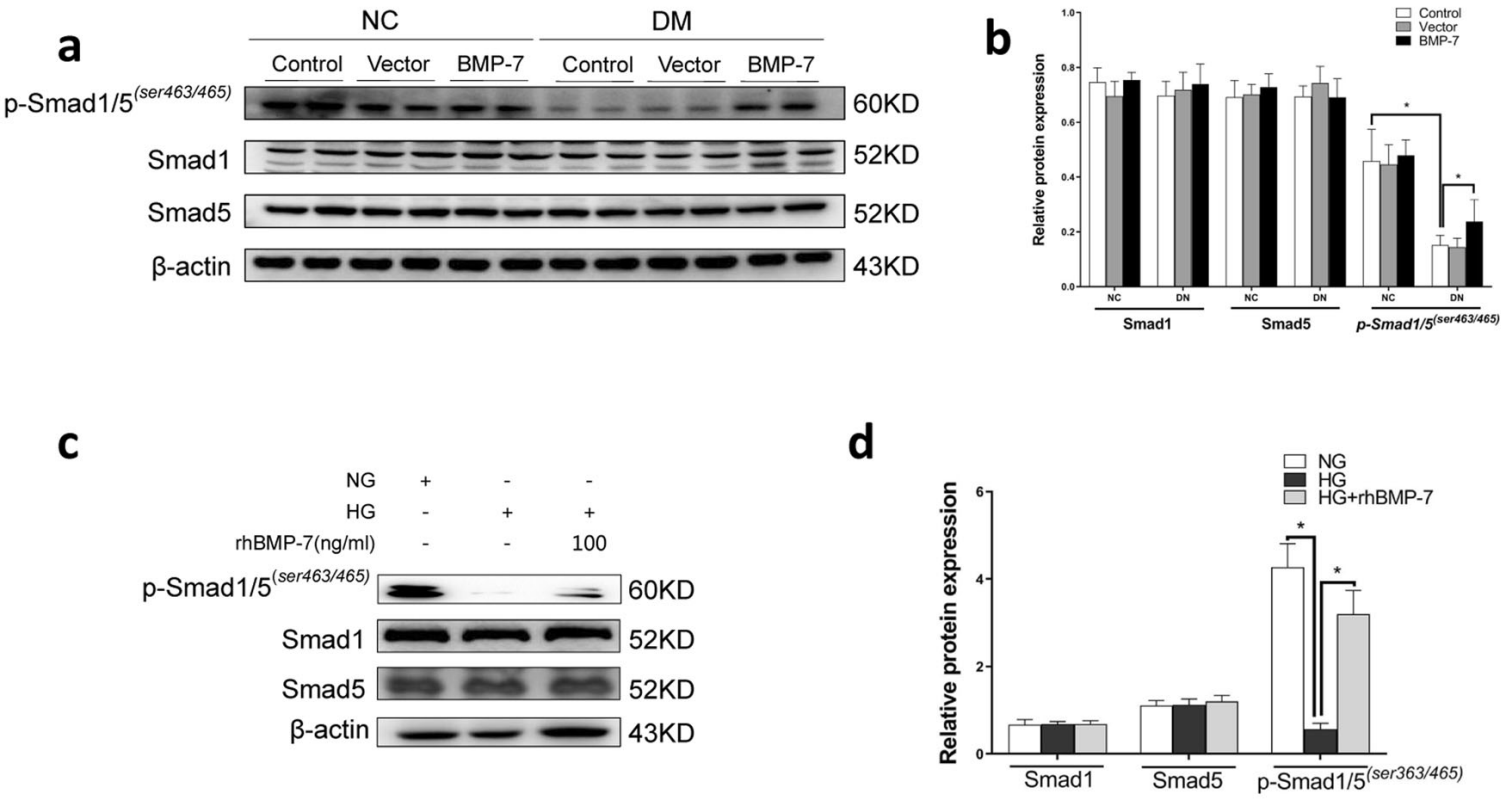
BMP-7 preserved the activity of p-Smad1/5 in vivo and in vitro. **a** Western blotting and **b** quantitative data represent the expressions of p-Smad1/5^*(ser463/465)*^, Smad1, and Smad5 in the kidney tissues in different groups, *P < 0.05 (*n* = 6). **c** Western blot and **d** quantitative data represent the expressions of p-Smad1/5^*(ser463/465)*^, Smad1, and Smad5 in NRK-52E cells under different conditions, *P < 0.05 (*n* = 3).

To determine the role of the Smad1/5 pathway in BMP-7 restoring SnoN protein, we detected phosphorylation of Smad1/5 (p-Smad1/5^(*ser463/465)*^) in vivo and in vitro. As shown in Fig. 5a, b, p-Smad1/5^(*ser463/465)*^ level was reduced during the development of DKD in the control group and vector group in vivo. In contrast, in diabetic mice injected with *OE-BMP-7* plasmid, p-Smad1/5^(*ser463/465)*^ level was partially elevated compared to the control group or vector group in diabetic mice. Similar to the in vivo results, a significant decrease was detected in p-Smad1/5^(*ser463/465)*^ level in NRK-52E cells exposed to high-glucose (Fig. 5c, d). As expected, BMP-7 maintained p-Smad1/5^(*ser463/465)*^ level in NRK-52E cells under high-glucose co-treatment with rhBMP-7. The results suggested that the function of BMP-7 was implemented through the Smad1/5 pathway in vivo and in vitro.

**Fig. 6:**
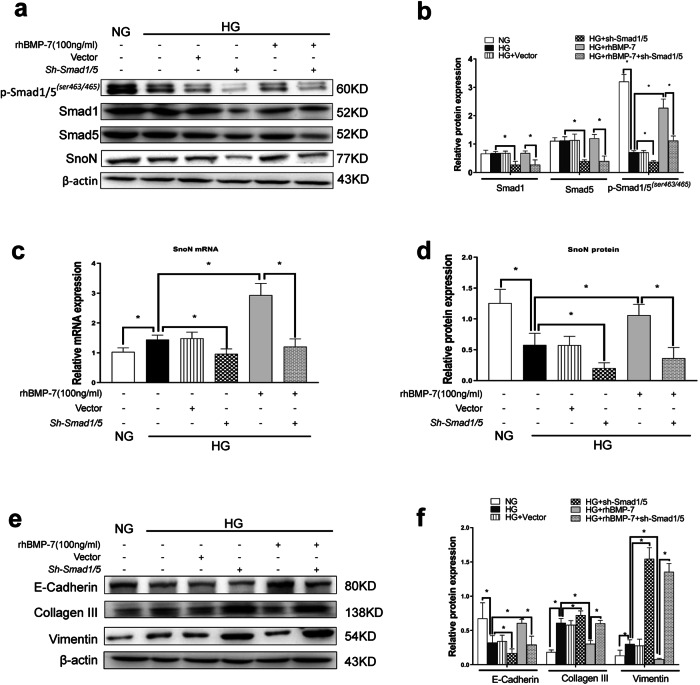
Inhibition of Smad1/5 expression lessened BMP-7 induced SnoN protein and the renoprotection of BMP-7 in renal tubular interstitial fibrosis. Knockdown Smad1/5 by transfection of *Smad1/5shRNA(sh-Samd1/5)* plasmid. Representative **a** Western blot and quantitative data were revealed the expression of **b** p-Smad1/5^*(ser463/465)*^, Smad1, Smad5, and **d** SnoN in NRK-52E cells under different conditions. **c** Graphical presentations show the relative abundance of *SnoN* mRNA after normalization with *β-actin* mRNA analyzed by RT-qPCR. Representative **e** Western blot and **f** quantitative data were revealed the expression of E-cadherin, Collagen-III, and Vimentin in NRK-52E cells under different conditions, *P < 0.05 (*n* = 3).

To explicate the patterns and the molecular mechanism involved in the regulation of SnoN expression in DKD, the expression of Smad1/5 in NRK-52E cells was knocked down by co-transfecting *Smad1shRNA* and *Smad5 shRNA* plasmids (*sh-Smad1/5*) (Supplementary Fig. 3a, b). The results of RT-qPCR and Western blotting confirmed that *Smad1shRNA* and *Smad5 shRNA* plasmids knocked down the expressions of Smad1 and Smad5, resulting in the decrease in *Smad1* and *Smad5* mRNA (Supplementary Fig. 3c, d) and protein levels and also in p-Smad1/5^(*ser463/465)*^ protein level (Fig. 6a, b). After the protein levels and activity of Smad1/5 were impaired, the simultaneous reduction in *SnoN* mRNA and protein levels was observed in NRK-52E cells in high glucose milieu (Fig. 6c, d). Furthermore, the effects of BMP-7 on the upregulated *SnoN* mRNA and protein levels were almost eliminated in NRK-52E cells under high-glucose co-treatment with rhBMP-7. In agreement with a previous report, Smad1/5 deficiency enhanced ischemia-induced TIF, which exhausted the antifibrotic effects of BMP-7 [20]. In addition, the results of Western blotting indicated that Smad1/5 deficiency enhanced the expressions of Vimentin and Collagen-III, reduced the expression of E-cadherin in NRK-52E cells under high-glucose conditions, and abolished BMP-upregulated E-cadherin expression and BMP-7-prevented Vimentin and Collagen-III in NRK-52E cells under high-glucose co-treatment with rhBMP-7 (Fig. 6e, f).

**Fig. 7:**
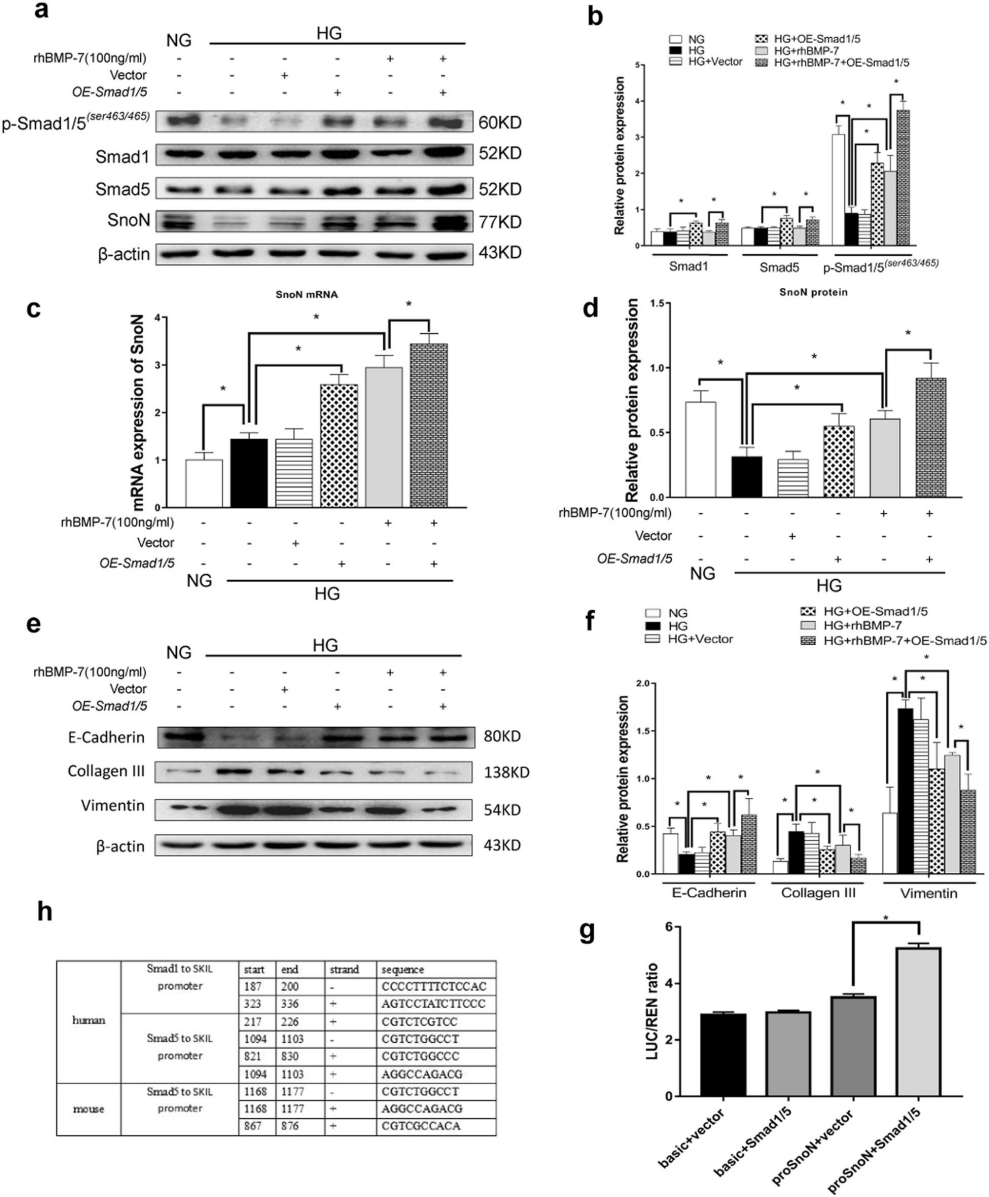
Augment of Smad1/5 expression enhances BMP-7 induced SnoN protein and the renoprotection of BMP-7 in renal tubular interstitial fibrosis. Increase Smad1/5 in NRK-52E cells by transfection of *Overexpression-Smad1/5(OE-Samd1/5)* plasmid. Representative **a** Western blot and quantitative data were revealed the expression of **b** p-Smad1/5^*(ser463/465)*^, Smad1, Smad5, and **d** SnoN in NRK-52E cells under different conditions. **c** Graphical presentations show the relative abundance of *SnoN* mRNA after normalization with *β-actin* mRNA analysised by RT-qPCR. Representative **e** Western blot and **f** quantitative data were revealed the expression of E-cadherin, Collagen-III, and Vimentin in NRK-52E cells under different conditions, *P < 0.05 (*n* = 3). **g** The binding site between Smad1/5 and *SKIL* promoter predicted by Jaspar. **h** Luciferase detected the binding of Smad1/5 to *SKIL* promoter in HEK 293 T cells.

On the contrary, co-transfecting with overexpressed *Smad1* and *Smad5* plasmids *(OE-Smad1/5)* (Supplementary Fig. 4a, b) increased the expressions of *Smad1* and *Smad5* mRNA (Supplementary Fig. 4c, d) and also p-Smad1/5^(*ser463/465)*^ protein level (Fig. 7a, b) in NRK-52E cells in a high-glucose milieu with or without BMP-7. Both *SnoN* mRNA and protein levels were also induced in NRK-52E cells co-transfected with *OE-Smad1/5* plasmids in high glucose milieu (Fig. 7c, d). Similarly, elevated *SnoN* mRNA and protein levels were detected in NRK-52E cells co-transfected with OE-Smad1/5 plasmids under high-glucose conditions with rhBMP-7. As illustrated in Fig. 7e, f, Smad1/5 overexpression upregulated E-cadherin expression and downregulated Vimentin expression in NRK-52E cells co-transfected with OE-Smad1/5 plasmids in high-glucose milieu, compared to the HG group. In addition, rhBMP-7 inhibited Collagen-III expression in NRK-52E cells co-transfected with *OE-Smad1/5* plasmids under high-glucose conditions with rhBMP-7.As shown in Fig. 7g, we transfected the *SKIL* promoter sequence with firefly luciferase(Supplementary Fig. 4e–g) in 293 T cells cultured in vitro, and then transfected *OE-Smad1/5* plasmids, the fluorescence signal in the cells was significantly higher than that in the proSnoN+vector group. Taken together, BMP-7 activated the Smad1/5 pathway to promote gene transcription and synthesis of *SnoN*.

**Fig. 8:**
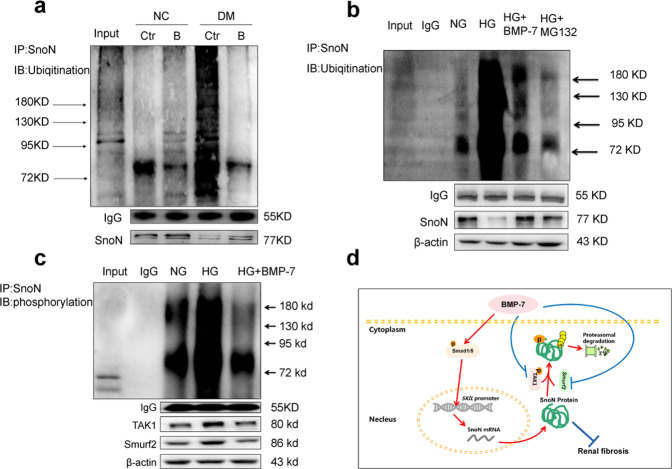
BMP-7 modulates ubiquitination of SnoN in renal tubular cells during DN. Immunoprecipitation shows the phosphorylation and ubiquitination of SnoN in the kidney tissues and NRK-52E cells in different groups. **a** Input:whole renal tissue lysis from NC group NC + Ctr:vehicle injection in normal mice;NC + B:OE-BMP-7 plasmid injection in normal mice;DM + Ctr:vehicle injection in diabetic mice;DM + B:OE-BMP-7 plasmid injection in diabetic mice. **b** Input:whole protein lysis from the NRK-52E cells;IgG:the negative control group of IP was treated with IgG antibody;NG:normal-glucose control group;HG:high-glucose control group;HG + BMP-7:high glucose+BMP-7(200 ng/mL rhBMP-7);HG + MG132:high glucose+MG132(5 μmol/L). **c** Input:whole protein lysis from the NRK-52E cells;IgG:the negative control group of IP was treated with IgG antibody;NG:normal-glucose control group;HG:high-glucose control group;HG + BMP-7:high glucose+BMP-7(200 ng/mL rhBMP-7). **d** Schematic image of the molecular mechanisms involved in the role of BMP-7 in restoring SnoN protein level.BMP-7 upregulates SnoN mRNA and protein levels by activating the classical Smad1/5 pathway to refrain from the partial EMT of RTECs and the deposition of ECM in DKD-induced renal fibrosis.

As described previously, the SnoN protein level was tightly regulated in the aspects of transcriptional activation and protein stability [11, 19]. Herein, we detected the ubiquitination levels of SnoN protein in NRK-52E cells in different milieu and kidneys of mice in different groups by immunoprecipitation. As a result, SnoN protein level decreased and ubiquitination of SnoN significantly increased in the DM group in vivo (Fig. 8a). However, the phenomenon was reversed in diabetic mice injected with the OE-BMP-7 plasmid. In vitro, the ubiquitination of SnoN protein was significantly elevated after high-glucose stimulation. Besides, the ubiquitination of SnoN protein decreased in HG + BMP-7 group compared to that in the HG group. Furthermore, proteasome inhibitor MG132 also restored SnoN protein level by preventing the degradation of SnoN. Compared with HG + MG132 group, the SnoN protein level was still higher in the HG + BMP-7 group (Fig. 8b), which indicated that BMP-7 reduced the degradation of SnoN by influencing Smad1/5, while the main factor should be the increase of synthesis of SnoN protein. In order to explore the molecular mechanism of BMP-7 preventing the ubiquitination and degradation of SnoN, we observed the expression of TGF-β-activated protein kinase 1 (TAK1) and Smad ubiquitination regulatory factor 2(Smurf2), which can regulate the phosphorylation and ubiquitination of SnoN, and the phosphorylation level of SnoN protein after stimulation by high glucose and BMP-7. The results showed that BMP-7 downregulated the expression of TAK1 and Smurf2, reduced the phosphorylation and ubiquitination of SnoN protein, and thus restored SnoN protein level (Fig. 8c).

## Discussion

In the present study, we found that exogenous BMP−7 could repair the renal injury in diabetic mice by reducing ACR, thickening of the glomerular basement membrane, suppressing tubular atrophy, infiltration of inflammatory cells, inhibiting phenotypic changes of proximal tubular epithelial cells, and secreting ECM mediated by high-glucose conditions. The protective effects of BMP−7 against high glucose-mediated fibrosis could be achieved by activating the Smad1/5 pathway, further upregulating *SnoN* mRNA level, as well as reducing the degradation of SnoN protein to restore the protein level.

Chronic kidney disease (CKD) derived from various diseases at end-stage renal failure are mainly characterized by TIF [2], including activation of tubular, production of fibrosis-promoting factors, increased ECM production, and reduced number of intact nephrons (refs. [5, 21]). During renal fibrosis, BMP-7 plays a major role in inhibiting TIF with respect to decreasing apoptosis, maintaining tubular epithelial phenotype, and inhibiting ECM synthesis (refs. [22–24]). Previous studies and the current study confirmed that the high-glucose milieu induced a progressive decrease in *BMP-7* mRNA and protein levels in kidney tissue, which was negatively correlated with renal fibrotic lesions (refs. [11, 12, 20]). Herein, we injected the *OE-BMP-7* plasmid into diabetic mice to upregulate the expression of BMP-7 in renal tissue (Fig. 1). Although exogenous BMP-7 did not mend the metabolism of diabetic mice (blood glucose, TGs, and TC), it markedly reduced the levels of urinary microalbumin/urinary creatinine, improved the thickening of glomerular basement membrane and mesangial hyperplasia, and reduced renal tubular atrophy and tubule interstitial widening. Moreover, BMP-7 reduced the interstitial Collagen-III and α-SMA positive staining of RTECs, decreased Vimentin expression, and promoted E-cadherin expression (Fig. 2). BMP-7 ameliorates DKD by preventing glomerulosclerosis reversing diabetic kidney hypertrophy and restoring glomerular filtration rate (GFR) in the progression of DKD [25–29]. EMT is a major source of activated myofibroblasts, although RTECs have not been proved to be transformed into activated myofibroblasts through the basement membrane, phenotypic changes may impair the reabsorption ability of RTECs and G2 cell cycle arrest. In addition, the secretion of a large number of profibrotic cytokines activates mesenchymal fibroblasts and immune cells, mediating inflammatory responses and direct synthesis of ECM [30, 31]. As a result, the tubular basement membrane was thickened and the renal interstitium was widened.

BMP-7 and its membrane receptors are highly expressed in renal distal tubular epithelial cells, while only the receptors are expressed in renal proximal tubular epithelial cells [8, 13, 22]. Several studies demonstrated that BMP-7 exerts protective effects on renal proximal tubular epithelial cells [11, 32, 33]. In our in vitro experiments, rhBMP-7 restored E-cadherin expression, and inhibited Collagen-III and Vimentin expressions, suggesting that BMP-7 could prevent high glucose-mediated partial EMT in RTECs (Fig. 3). However, the specific mechanism by which BMP-7 counteracts the effect of high glucose on fibrosis has not yet been clarified. Several studies attempted to find clues that BMP-7 is primarily responsible for the inhibition of TGF-β1 signaling [8, 13]. TGF-β signaling is also a nodal point integrating the fibrogenic actions of a variety of factors, such as AngII, high glucose, and connective tissue growth factor(CTGF) [34]. Exogenous BMP-7 ameliorates the expression of profibrotic genes *TGF-β1*, *Vimentin, and FN* in the aortae of mice with chronic uremia [7]. To date, only a few studies have revealed that BMP-7 can specifically inhibit TGF-β1 and hypoxia effects in RTECs via upregulation of PI3K inhibitor phosphatase and tensin homolog (PTEN) and Smad7 [12, 20]. This study showed that the effects of BMP-7 could be achieved by upregulating the level of SnoN protein and restricting the transmission of the TGF-β1 signaling pathway, while the knockdown of SnoN protein significantly reduced the BMP-7 inhibitory effect on PAI-1 transcription [14]. In addition, Luo et al. speculated that BMP-7 restored SnoN protein level in RTECs, which might be related to the inhibited degradation. According to our study, we further found that BMP-7 restored SnoN protein level by upregulating its mRNA level in RTECs cultured in high-glucose conditions (Fig. 4).

The nuclear transcriptional repressor SnoN is a nuclear protein encoded by a proto-oncogene that belongs to the Ski family, and SnoN regulates the TGF-β1/Smad signaling pathway [15, 16]. SnoN enters into the nucleus to limit the formation of heterologous complexes of p-Smad2/3 with Smad4 and represses the complex binding to TGF-β1 target genes [16]. Moreover, TGF-β1 can strongly upregulate the transcription of SnoN and promotes the ubiquitin degradation of SnoN protein, resulting in a decrease in the level of SnoN protein [11, 17]. Our previous study revealed that TGF-β1, which is highly expressed in RTECs, induces *SnoN* mRNA expression during DKD development [11, 18]. In addition, TGF-β1 activates TAK1, mediating SnoN protein phosphorylation to impair protein stability [19]. After the increase of the expressions of Smurf2 and Arkadia, the degradation of SnoN protein was enhanced via the activated TGF-β1/Smad pathway [35, 36]. In the present study, we found that BMP-7 promoted the upregulation of *SnoN* mRNA in a high-glucose medium, increased the SnoN protein level, maintained the phenotype of RTECs, and inhibited Collagen-III synthesis. These findings were supported by the reduced E-cadherin expression and inhibited expressions of Collagen-III and Vimentin in cells after transfecting into *sh-Skil* plasmid co-treated with BMP-7 (Fig. 4).

BMPs activated receptors mediate intracellular signaling of BMPs through Smad1/5 and non-Smad pathways [8, 13, 22]. In addition, a small molecule AA-123 that mimics BMP-7 activity by activating ALK3 signaling reproduces the antifibrotic activity of recombinant BMP-7 in murine models of renal fibrosis [37]. Thus, BMP-7 exerts its renal protective effects through classic Smad1/5 pathway. Furthermore, we observed the increased p-Smad1/5 *(ser463/465)* expression in kidney tissue of *OE-BMP-7* plasmid-treated DM mice and NRK-52E cells co-treated with rhBMP-7 in high-glucose conditions (Fig. 5). Knockdown of smad1/5 could significantly affect the effects of rhBMP-7 on the upregulation of SnoN mRNA and protein levels, as well as its anti-partial EMT (Fig. 6). Conversely, with co-transfecting *overexpression-Smad1* and *overexpression-Smad5* plasmids into NRK-52E cells cultured in high-glucose, the effect of rhBMP-7 on upregulating SnoN mRNA and protein levels was further boosted, And it is the effect produced by the combination of Smad1/5 and the promoter of *SKIL*, and the protective effect against partial EMT was enhanced (Fig. 7). This finding was supported by the administration of BMP-9 into pulmonary vascular smooth muscle cells and the binding of Smad1/5 to the *SKI* gene [38]. However, the level of SnoN protein was also influenced by the degradation of the ubiquitin-proteasome system, and Luo et al. speculated that the expression of SnoN protein in RTECs by BMP-7 was related to the inhibited degradation [14, 16]. Therefore, we examined the ubiquitination of SnoN protein in vivo and in vitro, with an increase in the ubiquitination of SnoN protein level in NRK-52E cells cultured in high-glucose milieu and kidneys of diabetic mice, in which BMP-7 limited the phosphorylation and ubiquitination of SnoN protein in cells and kidneys (Fig. 8a–c).

In conclusion, we speculated that BMP-7 could upregulate *SnoN* mRNA level by activating the classical Smad1/5 signaling pathway, and it also could reduce the ubiquitination of SnoN protein, finally restoring the SnoN protein level to inhibit hyperglycemia-mediated renal tubular epithelial fibrosis (Fig. 8d). However, further researches are required to indicate how BMP-7 can inhibit the ubiquitination-mediated degradation of SnoN protein.

## Materials and methods

### Animal model

The number of experimental animals was determined to be 36 mice by the evaluation of the degree of freedom (E) of analysis of variance and the estimation of modeling success rate. A total of 36 healthy C57BL/6 mice (weight: 18 ± 2 g) were obtained from Beijing Si Bei Fu Bioscience Co., Ltd (Beijing, China) and housed in the Animal Center of Guizhou Medical University (Guizhou, China). This study was conducted in accordance with the guidelines of the National Health and Medical Research Council of China for the care and use of animals for scientific purposes. Diabetic mice were produced by injecting 0.01 mol/L streptozotocin (STZ, prepared with sterile citric acid–sodium citrate buffer, pH 4.5; Sigma-Aldrich, MO, St. Louis, USA).intraperitoneally (i.p.) at a dose of 55 mg/kg/d for 5 days. Fasting blood glucose levels of mice were detected after 72 h. The blood glucose level ≥16.7 mmol/L indicated that the mouse model of DM was established successfully. All mice were randomly divided into the diabetic group (DM group, *n* = 18), while normal control (NC) mice were age-matched (*n* = 18), and an equivalent volume of solvent was injected into each control rat. Mice were given a normal diet and unlimited drinking water. After confirming that the mice have diabetes for 6 weeks, the mice were divided into six groups: (i) vehicle (Ringer’s Solution:NaCl 0.147 M, KCl 4 mM, CaCl_2_ 2.5 mM) injection in normal mice (NC + Control); (ii) vector plasmids injection in normal mice (NC + Vector); (iii) OE-BMP-7 plasmid injection in normal mice (NC + BMP-7); (iv) vehicle injection in diabetic mice (DM + Control); (v) vector plasmid injection in diabetic mice (DM + Vector); (vi) OE-BMP-7 plasmid injection in diabetic mice (DM + BMP-7) (*n* = 6). The purpose of injecting exogenous OE-BMP-7 plasmids via hydrodynamic tail vein injection, a method to rapidly deliver naked plasmids to mouse tissues, is to enable the high expression of BMP-7 in the mouse kidney. Mice were injected respectively through the tail vein with vehicle-Ringer’s Solution 1.5 ml as control, 1.5 ml Ringer’s Solution with 15 mg vector plasmids as Vector treatment, or OE-BMP-7 plasmids as BMP-7 treatment every week for 6 weeks [27]. At the end of 12 weeks, all mice were sacrificed. Their 24-h urine was collected in metabolic cages before sacrificing the animals. The mice were fasted for 6–8 h before anesthetizing with pentobarbital sodium. The femoral artery was punctured, and serum was separated in blood samples at 4000 rpm for 10 min by centrifugation at 4 °C. Urine and serum were stored at –20 °C for measuring the urine protein and biochemical indices. Subsequently, the kidneys of the mice were harvested; one part of the kidney was fixed with 4% paraformaldehyde for embedding tissue sections into paraffin, and the other part was snap-frozen in liquid nitrogen and stored at –80 °C for RNA and protein extractions.

### Evaluation of biochemical markers

The oxidase method was used to measure serum glucose (Nanjing Jiancheng Bioengineering Institute, Nanjing, China) [39]. Immune turbidimetry and the Benedict–Behre method was used to measure urinary microalbumin/urinary creatinine. Urine microalbumin measurement used the enzyme-linked immunosorbent method (Elabscience Biotechnology Co., Ltd., Wuhan, China), and urine creatinine measurement was performed using sarcosine oxidase method (Nanjing Jiancheng Bioengineering Institute). The levels of total cholesterol (TC) and triglycerides (TGs) were determined using enzymatic assay kits, according to the manufacturer’s instructions (Nanjing Jiancheng Bioengineering Institute).

### Histopathological analysis

Kidneys fixed in paraformaldehyde were embedded in paraffin, and transverse sections (4 µm) were sliced for hematoxylin-eosin (H&E) staining and Masson’s trichrome staining. The morphology of renal tissue was observed under a light microscope, and renal tissue fibrosis was observed by H&E and Masson’s trichrome staining. H&E’s renal tubular lumen area and Masson’s trichrome-positive area were analyzed relative to the whole area from six random fields (200×).

### Cell culture and treatments

RTECs (NRK-52E cells, Conservation Genetics CAS Kunming Cell Bank, Kunming, China) were cultured in normal-glucose (5.5 mmol/L glucose) in a Dulbecco’s modified Eagle’s medium (DMEM; HyClone, Logan, UT, USA) supplemented with 5% fetal bovine serum (FBS; Invitrogen, Carlsbad, CA, USA) and HEK 293 T cells were cultured in Ham’s F 12 nutrient medium (F-12; HyClone, Logan, UT, USA) supplemented with 10% fetal bovine serum (FBS; Invitrogen, Carlsbad, CA, USA) in an incubator with 5% CO_2_ at 37 °C. The cells were growth-arrested in a serum-free medium for 20 h and then treated with the following media: (1) normal-glucose control group (NG) (5.5 mmol/L glucose); (2) high-glucose control group (HG) (5.5 mmol/L glucose + 19.5 mmol/L d-glucose); (3) high glucose + BMP-7 (12.5, 25, 50, 100, and 200 ng/mL rhBMP-7); and (3) high glucose + MG132(5 μmol/L). Cells in each group were cultured for 48 h for further study.

### Transfection

The plasmid was extracted according to the manufacturer’s protocol. Transfection was conducted when the NRK-52E cells reached 50–60% confluency. A DMEM culture medium (no serum, no antibiotics), polyethyleneimine, and plasmid (ski-shRNA (*sh-Skil)*, overexpressed *Smad1* plasmid, and overexpressed *Smad5* plasmid *(OE-Smad1/5), Smad1shRNA* plasmid, and *Smad5 shRNA* plasmid (*sh-Smad1/5*), Supplementary Figs. 2–4) were placed in a sterile environment for 20 min. The culture medium was discarded. The liquid was put in an Eppendorf tube and blended, and the cells were then incubated at 37 °C in the presence of 5% CO_2_ for 6 h. Afterward, the DMEM medium (no serum, no antibiotics) was added, and the cells were incubated at 37 °C overnight in the presence of 5% CO_2_. Then, the culture medium was discarded, and the cells were washed with phosphate-buffered saline (PBS). The culture media of different treatments were added to the cells as needed, and the system was incubated at 37 °C in the presence of 5% CO_2_ for 48 h.

### Immunohistochemistry and immunofluorescence (IF) staining

The biotin-streptavidin-peroxidase method (ZSBio, Beijing, China) was used to stain tissue sections incubated with mouse-anti-α-SMA (1:100; Santa Cruz Biotechnology Inc., Dallas, TX, USA) and antibodies to show the distribution and expression of the protein in kidney tissues. For the negative control, PBS was substituted for the primary antibody. The secondary antibodies were affinity-purified biotinylated anti-mouse immunoglobulin G (IgG), followed by streptavidin-peroxidase complex. The signals were visualized using 3,3′-diaminobenzidine (DAB) chromogen. The tissue sections were counterstained with H&E.

Cells cultured on coverslips were twice washed with cold PBS and fixed with cold methanol: acetone (1:1) for 10 min on ice. Then, 3-μm-thick cryosections of renal tissue were prepared for IF staining. After extensive washing, the sections were blocked with bovine serum antigen for 30 min at room temperature and incubated with the following specific primary antibodies: rabbit-anti-BMP-7 (1:200; Proteintech, Wuhan, China), rabbit-anti-SnoN (1:200; Proteintech) rabbit-anti-E-cadherin (1:200, Santa Cruz Biotechnology, Inc.), mouse-anti-α-SMA (1:100), and rabbit-anti-Collagen-III (1:200, Sigma-Aldrich) overnight at 4 °C. After that, the cells were stained with cyanine-5 (Cy5)-conjugated goat-anti-rabbit IgG (1:400; Protientech), or cyanine-3 (Cy3)-conjugated goat-anti-rabbit IgG (1:400; Protientech), and cyanine-3(Cy3)-conjugated goat-anti-mouse IgG (1:400; Protientech). After washing, the sections were stained with 4′,6-diamidino-2-phenylindole (DAPI) to visualize the nuclei and observed under an inverted fluorescence microscope. Six visual fields (400×) were randomly selected in each group, and the gray value obtained by positive staining was analyzed by the ImageJ software for statistics.

### Western blotting

Protein expression in renal tissue and cultured cells was analyzed by Western blotting. Cells were lysed in ice-cold lysis buffer, and protein concentration was determined using the BCA Protein Assay Kit (Beyotime). The protein samples were separated by sodium dodecyl sulfate-polyacrylamide gel electrophoresis (PAGE) and electrotransferred to polyvinylidene difluoride (PVDF) membrane (Millipore, Germany). Subsequently, the membranes were blocked with 5% non-fat milk for 1 h at room temperature and probed with primary antibodies, such as rabbit-anti-BMP-7 (1:1000), rabbit-anti-Smad1 (1:1000; Proteintech), rabbit-anti-Smad5 (1:1000; Proteintech), rabbit-anti-Phospho- Smad1/5^*(Ser463/465)*^ (1:1000, Cell Signaling Technology), rabbit-anti-E-cadherin (1:1000), rabbit-anti-Vimentin (1:1000; Santa Cruz), rabbit-anti-Collagen-III (1:1000), rabbit-anti-SnoN (1:1000), and rabbit-anti-β-actin (1:400; Proteintech), for 16 h at 4 °C. The signals were detected by chemiluminescence after incubation with appropriate secondary antibodies (). Images were acquired using the Bio-Rad gel imaging system (Bio-Rad, CA, USA), and the intensities of the immunoreactive bands were quantified using the Quantity One 4.6 software (Bio-Rad).

### Immunoprecipitation

Total protein lysates were prepared in 50 mM Tris-HCl (pH 7.4) 0.2 M NaCl, 2 mM ethylene diamine tetraacetic acid (EDTA), 0.5% NP-40, 50 mM NaF, 0.5 mM Na_3_VO_4_, 20 mM sodium pyrophosphate, 1 mM phenylmethanesulfonyl fluoride (PMSF), 10 µg/mL aprotinin, 10 µg/mL leupeptin, 1 mM dithiothreitol (DTT), and 150 mM NaCl. Co-immunoprecipitation was performed by adding Dynabeads-Ab complexes to an equivalent amount of protein lysates(5 μg/μl) and incubating the mixture overnight at 4 °C. Finally, the coimmunoprecipitate complexes were washed and subjected to Western blot analysis. The input group was the positive control group, which was the whole protein extracted from the cells.

### Real-time quantitative reverse transcription-polymerase chain reaction (RT-qPCR)

Total RNA in the rat renal cortex or NRK-52E cells was extracted using TRIzol (Tiangen, Dalian, China) method, according to the manufacturer’s protocol. First-strand cDNA was synthesized from 5 μg total RNA in renal tissues and 3 μg total RNA in NRK-52E cells using the RevertAid First-Strand cDNA Synthesis Kit (Fermentas, Lithuania) and stored at −20 °C. RT-qPCR was performed using iQSYBR GreenSupermix (Bio-Rad Laboratories Inc., Hercules, CA, USA).

After 45 cycles of amplification, data were collected to plot kinetic curves, and the Ct values were acquired. These steps were repeated three times, and the 2^–ΔΔCt^ method was used to calculate the mRNA level of the target genes and normalized to that of β-actin in each group. Primer sequences for quantitative PCR were shown in Supplementary Table S1.

### Luciferase reporter assay

The SnoN-promoter-luciferase reporter was constructed by Longqian Biotech (China). Actively proliferating HEK 293 T cells were trypsinized and seeded in plates at a suitable density for routine culture. Following 24 h of incubation, transfection was carried out with Lipofectamine 2000 (Invitrogen) as directed by the manufacturer for 48 h. This was followed by cell lysis and sample analysis with a Dual-Luciferase Reporter Assay System (E1960; Promega, USA). Firefly and Renilla luciferase activities were measured, and the ratio of Firefly luciferase activity to that of Renilla luciferase was derived. Triplicate experiments were independently repeated three times.

### Statistical analysis

All data were expressed as mean ± standard deviation (${{{\bar{\mathrm X}}}}$ ± SD). SPSS 18.0 software (IBM, Armonk, NY, USA) was used to perform statistical analysis. Statistical analysis between the groups was performed using an unpaired Students *t*-test, while comparison among multiple groups was performed using one-way analysis of variance(ANOVA) followed by post hoc Bonferroni correction. *P* < 0.05 was considered statistically significant.

## Supplementary information

Supplementary Fig.1

Supplementary Fig.2

Supplementary Fig.3

Supplementary Fig.4

Supplementary Table S1

## Data Availability

All the data generated or analysed during this study are included in this published article and its supplementary files.
