# Supplementary figures and images for: BMP-7 ameliorates partial epithelial-mesenchymal transition by restoring SnoN protein level via Smad1/5 pathway in diabetic kidney disease
Source: Cell Death Dis. 2022 Mar 21;13(3):254. doi: 10.1038/s41419-022-04529-x (PMC8938433; doi:10.1038/s41419-022-04529-x)

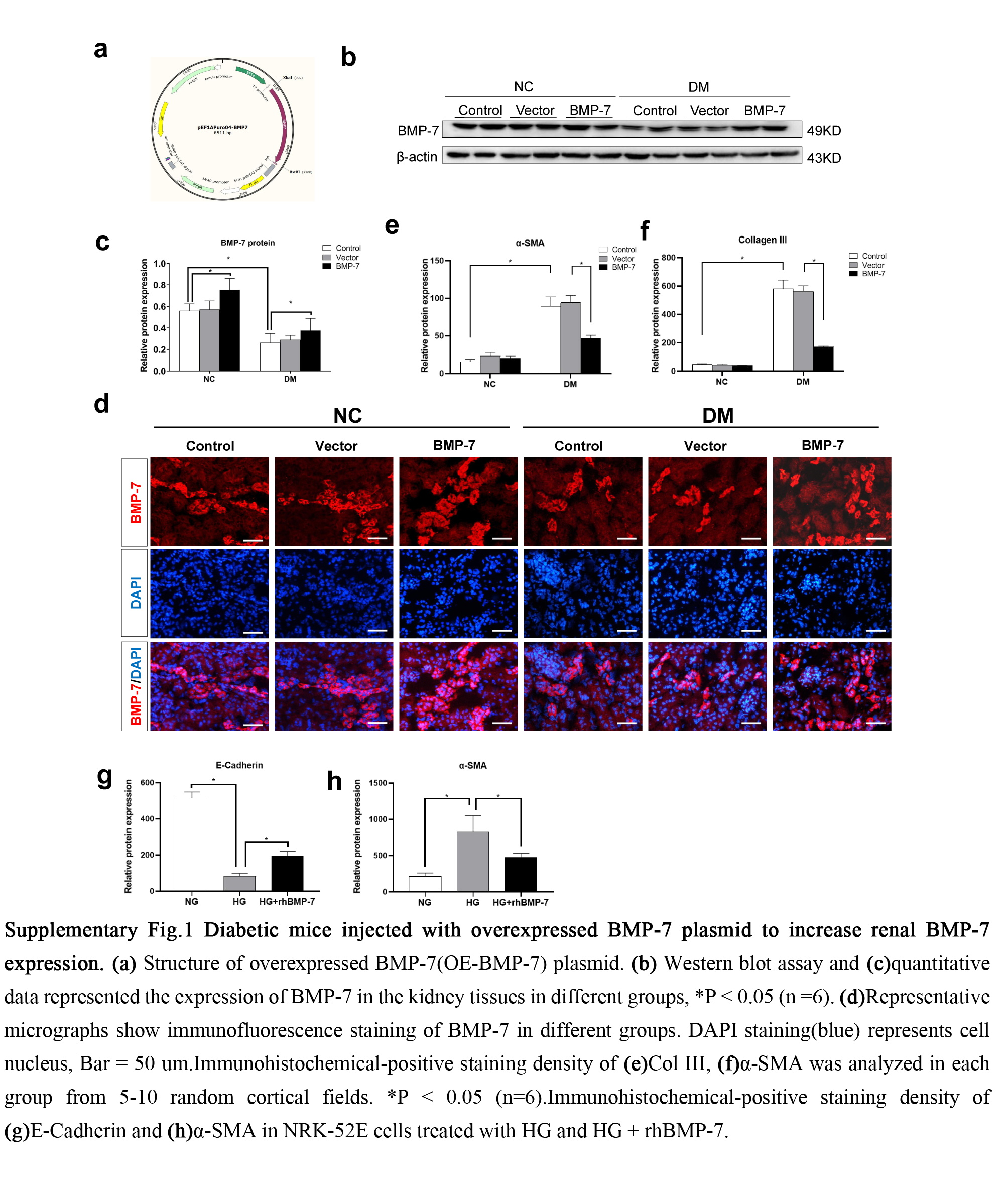

Supplement: Supplementary file 1 — Supplementary Fig.1 [file 41419_2022_4529_MOESM1_ESM.tif]

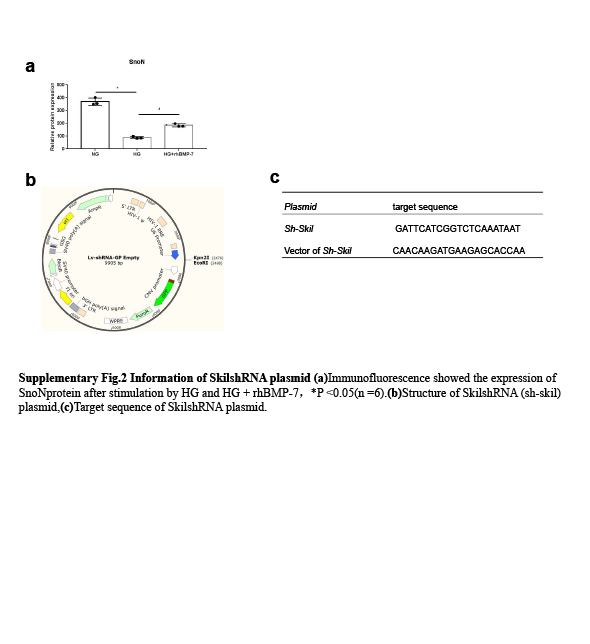

Supplement: Supplementary file 2 — Supplementary Fig.2 [file 41419_2022_4529_MOESM2_ESM.tif]

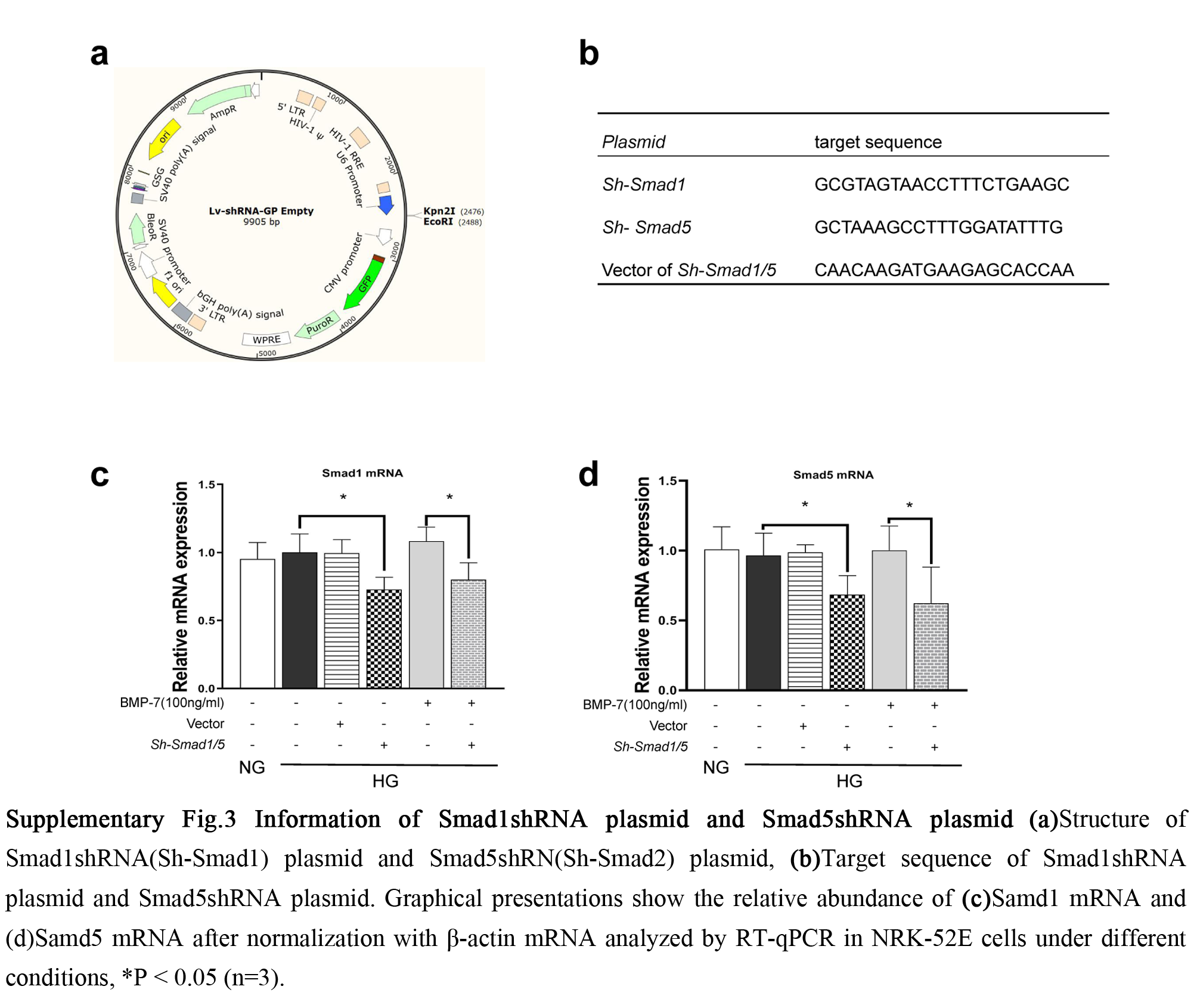

Supplement: Supplementary file 3 — Supplementary Fig.3 [file 41419_2022_4529_MOESM3_ESM.tif]

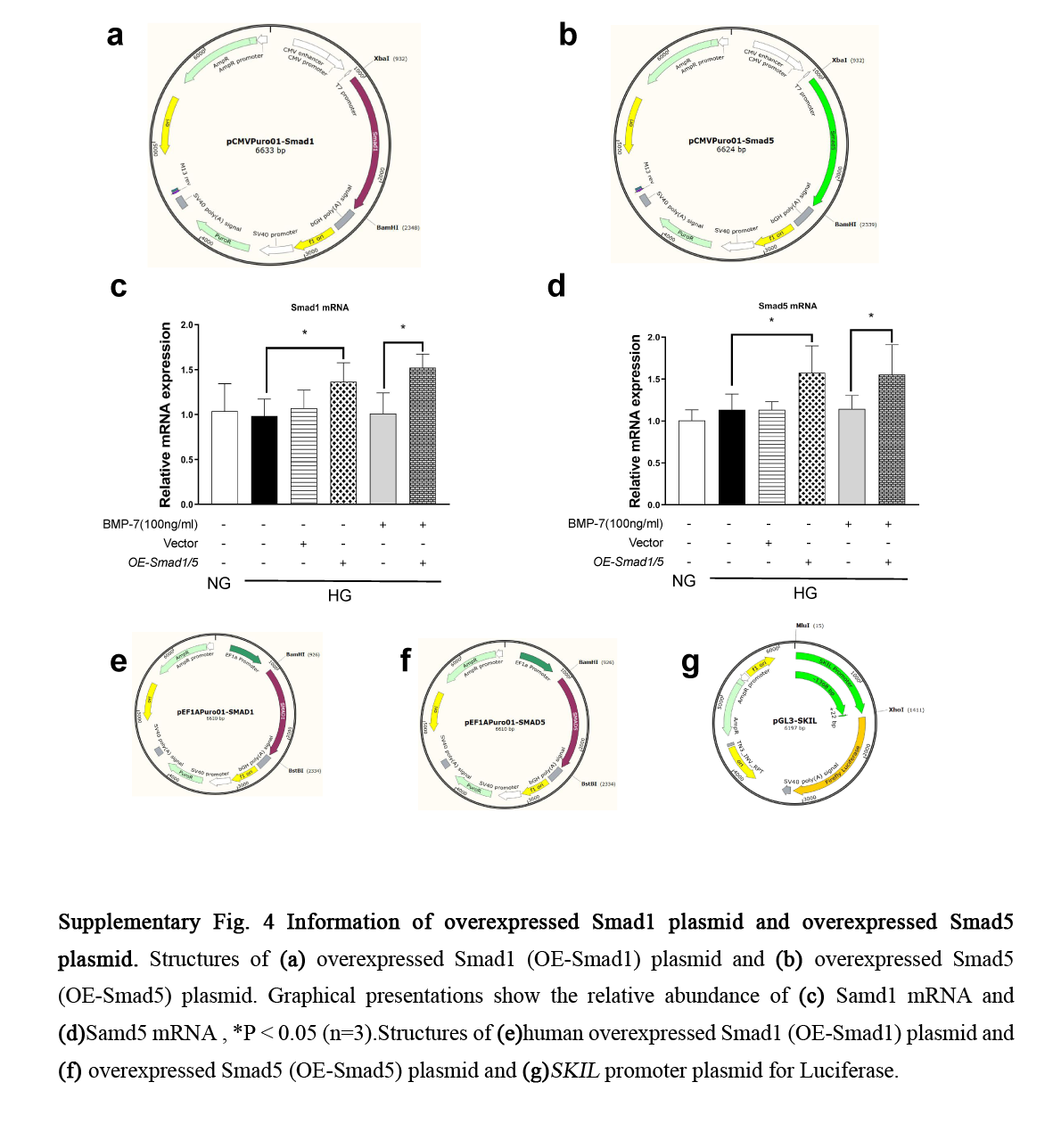

Supplement: Supplementary file 4 — Supplementary Fig.4 [file 41419_2022_4529_MOESM4_ESM.tif]
