# Supplementary material for: BMP-7 ameliorates partial epithelial-mesenchymal transition by restoring SnoN protein level via Smad1/5 pathway in diabetic kidney disease
Source: Cell Death Dis. 2022 Mar 21;13(3):254. doi: 10.1038/s41419-022-04529-x (PMC8938433; doi:10.1038/s41419-022-04529-x)
Supplement: Supplementary file 5 — Supplementary Table S1 [file 41419_2022_4529_MOESM5_ESM.docx]

**Table S1. S**equences of RT-qPCR primers used in this study

| **Primer** | **Sequences** |
| --- | --- |
| SnoN(Rat)  (NCBI Reference Sequence:XM_008760949.3) | Forward: 5′-TGTCTGAGAAACATGGTCACCTTCC -3′ |
|  | Reverse: 5′-AGGGAGCGTCGGGCTGAACATA-3′ |
| Actin(Rat)  (GenBank: V01217.1) | Forward: 5′- ACCACCATGTACCCAGGCAT-3′ |
|  | Reverse: 5′- CCGGACTCATCGTACTCCTG-3′ |
| Smad1(Rat)  (GenBank: AF067727.1) | Forward:5′- GCCACCATGAACTGAAGCCTCTG-3′ |
|  | Reverse:5′- TACTCGCTGTGCCTCGGAACC-3′ |
| Smad5(Rat)  (GenBank: AB010955.1) | Forward: 5′- GCTTCTGGCTCAGTCAGTCAACC-3′ |
|  | Reverse: 5′- ACGTCCTGTCGGTGGTACTCTG-3′ |
| SnoN(mice)  (GenBank: BC049934.1) | Forward: 5′-GTGTCTGGAGTGCTGTGGAA-3′ |
|  | Reverse: 5′-GCTGGGGTGTAAAAATGAATG-3′ |
| Actin(mice)  (GenBank: AY618569.1) | Forward: 5′-GCGGTGCACGATGGAGGGGC-3′ |
|  | Reverse: 5′-GGACCTGGCTGGCCGGGACC-3′ |
